# Continuous use of metformin can improve survival in type 2 diabetic patients with ovarian cancer: A retrospective study

**DOI:** 10.1097/MD.0000000000007605

**Published:** 2017-07-21

**Authors:** Shan-Bing Wang, Kai-Jian Lei, Jia-Pei Liu, Yu-Ming Jia

**Affiliations:** a Department of Oncology; b Laboratory Medicine, the Second People's Hospital of Yibin City, Yibin, Sichuan, China.

**Keywords:** diabetic, metformin, ovarian cancer, survival

## Abstract

Evidence indicates that type 2 diabetes may stimulate the initiation and progression of several types of cancer. Metformin, a drug most commonly used to treat type 2 diabetes, may inhibit cancer cell growth and reduce the risk of cancer. However, evidence of the antitumor effects of metformin on ovarian cancer is still limited.

In this study, we retrospectively examined the effects of metformin on ovarian cancer patients with diabetes at our institution.

We identified 568 consecutive patients who were newly diagnosed with ovarian cancer and treated between January 2011 and March 2014. Patients with International Federation of Gynecology and Obstetrics (FIGO) stage I to IV epithelial ovarian, fallopian, or peritoneal cancer were included. Patients with type 1 diabetes, incomplete records (including medication records) and any other cancer before their ovarian cancer diagnosis, as well as those diagnosed with diabetes more than 6 months after their ovarian cancer diagnosis, were excluded. Out of 568 patients, 48 (8.5%) patients with type 2 diabetes continuously used metformin, 34 (5.9%) patients with type 2 diabetes did not take metformin, 22 (3.9%) patients with type 2 diabetes discontinued metformin, and 464 (81.7%) ovarian cancer patients were nondiabetic controls. Longer progression-free survival (PFS) and overall survival (OS) were observed in ovarian cancer patients with diabetes who were taking metformin than in diabetic patients not taking metformin, diabetic patients who discontinued metformin, and nondiabetic ovarian cancer patients (*P* = .001). After adjusting for possible confounders, metformin use was associated with a lower risk for disease relapse [hazard ratio (HR) = 0.34; 95% confidence interval (CI): 0.27–0.67; *P* < .01] and disease-related death (HR = 0.29; 95% CI: 0.13–0.58, *P* *=* .03) among ovarian cancer patients with diabetes.

Metformin use may decrease the risk for disease recurrence and death in patients with ovarian cancer, but the drug treatment must be continuous.

## 1 Introduction

Ovarian cancer is the leading cause of gynecological cancer associated death in China and is the most common cause of cancer death in women.^[1]^ This is because over two-thirds of patients have progressed to late-stage disease [International Federation of Gynecology and Obstetrics (FIGO) stage III or IV] by the time of diagnosis.^[2]^ Although advances in primary surgery and adjuvant therapy have attained high rates of complete pathological response with modern management,^[3]^ the majority of ovarian cancer patients who present with late-stage disease will relapse within 18 months.^[4]^ Unfortunately, the currently available systemic therapy for recurrent ovarian cancer, such as cytoreductive surgery followed by combination chemotherapy, has limited efficacy.^[5]^ Consequently, we need to develop more effective therapies for ovarian cancer.

Type 2 diabetes has become increasingly prevalent worldwide; approximately 552 million people throughout the world will have diabetes by 2030.^[6]^ Emerging evidence from multiple studies and meta-analyses have reported that type 2 diabetes is associated with an increased incidence of and mortality from many cancers, such as hepatic cancer, colorectal cancer, and ovarian cancer.^[7]^ How diabetes causes cancer is not yet clear, but many studies have suggested that secondary hyperinsulinemia may induce or stimulate mitogenic processes through its cognate receptor or via the insulin-like growth factor-1 (IGF1) receptor.^[8,9]^ Furthermore, hyperglycemia can induce the emergence of oxidative stress, which may promote carcinogenesis.^[10]^

Metformin, a biguanide commonly used as a first-line pharmacotherapy for type 2 diabetes, may decrease the risk of several types of cancers.^[11,12]^ The in vitro anti-tumorigenic effects of metformin have been reported in cancers of the breast, prostate, and colon.^[13–16]^ Several retrospective studies have suggested that metformin use in patients with diabetes and concurrent breast or prostate cancer led to longer progression-free survival (PFS) and overall survival (OS).^[17–19]^ However, until now, only 3 retrospective studies have evaluated the relationship between metformin use and survival in patients with ovarian cancer, and their findings are inconsistent.^[20–22]^ In this study, we retrospectively examined the effects of metformin on ovarian cancer patients with diabetes at our institution.

## 2 Methods

### 2.1 Study population

We recruited 631 consecutive patients who were newly diagnosed with ovarian cancer and treated between January 2011 and March 2014 at the Second People's Hospital of Yibin. All patients with FIGO stage I to IV epithelial ovarian, fallopian, or peritoneal cancer were included. Among these patients, 112 had type 2 diabetes. Patients with type 1 diabetes, incomplete records (including medication records), and any other cancer before their ovarian cancer diagnosis as well as those diagnosed with diabetes more than 6 months after their ovarian cancer diagnosis were excluded. Finally, 571 patients were analyzed and divided into the following 4 groups: 48 patients with type 2 diabetes taking metformin (metformin group), 34 patients with type 2 diabetes not taking metformin (non-metformin group), 22 patients with type 2 diabetes who discontinued metformin more than 6 months before relapse (discontinued group), and 464 nondiabetic patients (nondiabetic group). In addition, there were 3 patients with type 2 diabetes who discontinued metformin less than 6 months before relapse (due to the small sample size, analysis of this group was prohibited). This retrospective study was approved by the Ethics Committee of the Second People's Hospital of Yibin.

The purpose of this retrospective study was to explore whether there was a difference in PFS and OS between ovarian cancer patients with type 2 diabetes and those without type 2 diabetes. Patient demographics and tumor characteristics, including age, smoking, body mass index (BMI), pathology, diagnosis of diabetes, antidiabetic medications, chemotherapy data (number of cycles, agents and administration approach), PFS, and OS, were obtained from medical records. Tumor recurrence or progression was defined as follows: evidence of the reappearance of the tumor by clinical assessment, new tumor lesions revealed by radiography, or a rising CA-125 more than twice the upper limit of normal.^[23]^ PFS was defined as the time from the date of diagnosis to the first recurrence of the disease or death. OS was defined as time from the date of diagnosis to the last known follow-up or death from any cause.

### 2.2 Statistical analysis

F-tests and Fisher exact tests were used to compare continuous data and categorical data. Kaplan–Meier estimates were used for the analysis of PFS and OS, and the survival curves of the 4 groups were compared with log-rank tests. A multivariate Cox proportional hazard model was used to estimate PFS and OS with adjustments for confounders, including age, histological subtype, grade, BMI, smoking, type of surgery, postoperative residual disease, and chemotherapy drug delivery approaches. All statistical analyses were performed using SPSS version 22.0 (IBM, Armonk, NY), and *P* values ≤.05 were considered statistically significant.

## 3 Results

### 3.1 Patient demographics and baseline clinical characteristics

From January 2011 to March 2014, 568 women were diagnosed with FIGO state I-IV ovarian cancer at our hospital. Among these patients, approximately 18.3% (104/568) of patients were documented to have diabetes, 70 out of 104 diabetic patients were recorded as using metformin at baseline, but 22 out of those 70 diabetic patients discontinued their metformin use due to inadequate glycemic control. In the metformin group, 27 patients were treated with 500 mg twice daily and 21 patients were treated with 1000 mg twice daily. Table 1 summarizes the patient demographics and tumor characteristics of the study. Baseline clinical features, including age, smoking, FIGO stage, histological subtype, and pathological grade, were not significantly different among the 4 groups (Table 1). The use of insulin was not different among the metformin group, the non-metformin group, and the discontinued group [13 (27.1%) vs 16 (47.1%) vs 10 (45.5%); *P* = .13]. Moreover, the rate of platinum agent used, the route of anticancer drug administration, and the number of chemotherapy cycles were similar among the 4 groups. The most frequently used drugs were carboplatin (74%) and paclitaxel (85%). The BMI of diabetic patients in the metformin group, the non-metformin group, and the discontinued group was higher than that of the patients in the nondiabetic group (26.2 vs 27.9 vs 26.4 vs 25.3 kg/m^2^; *P* *<* .03).

**Table 1:**
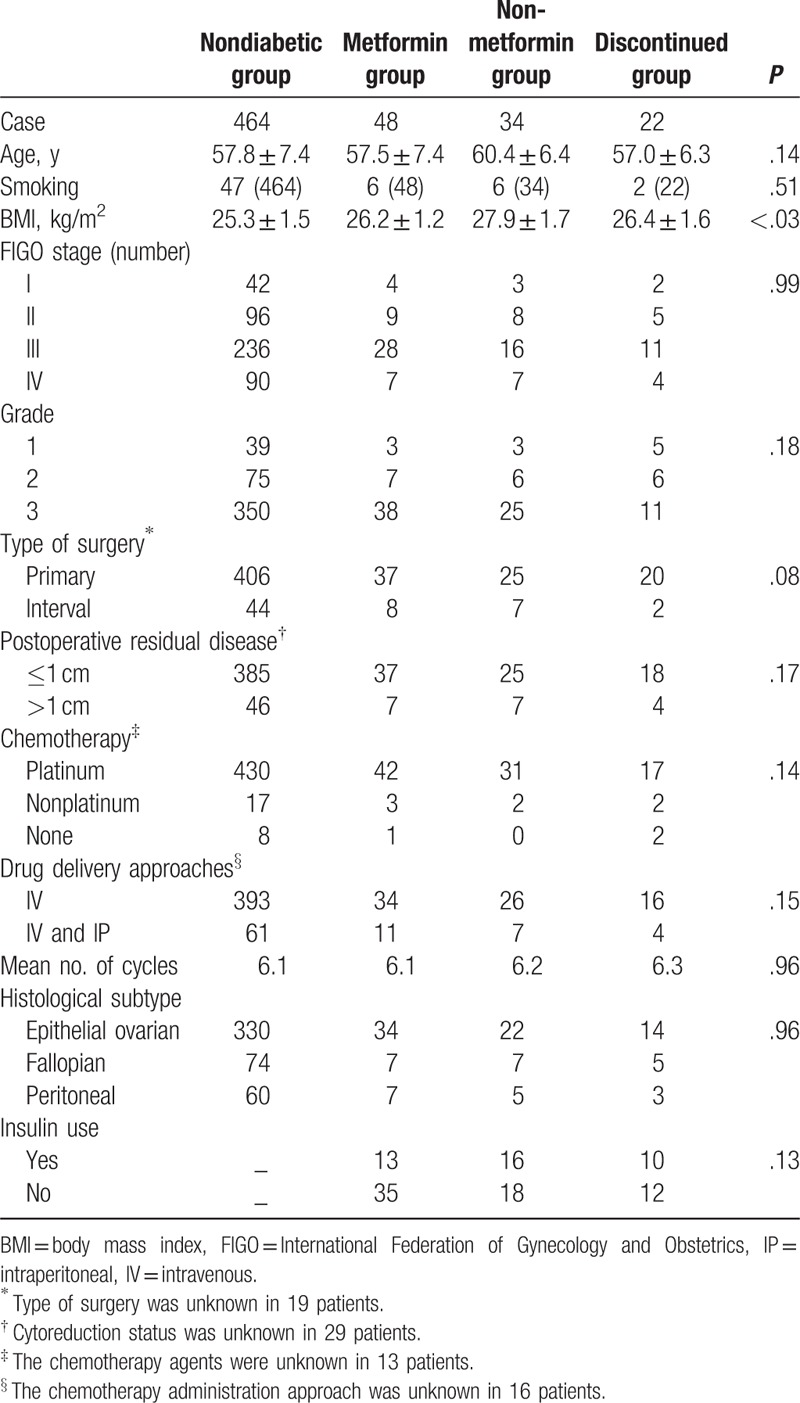
Patients demographics and baseline clinical characteristics.

### 3.2 Metformin use and survival analysis

**Figure 1:**
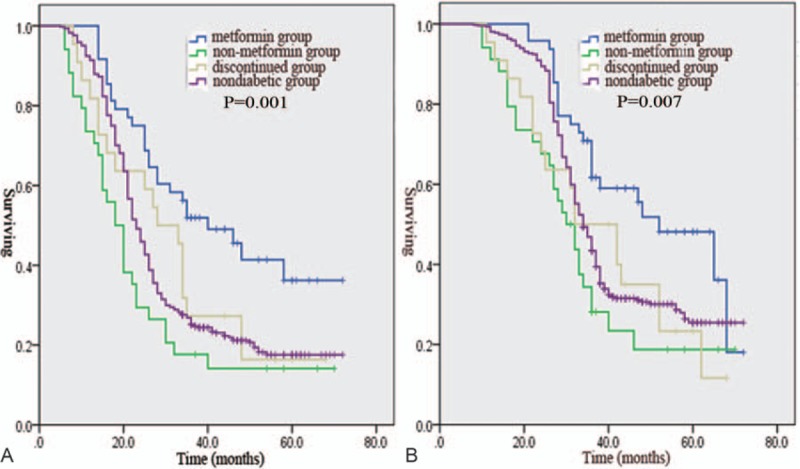
Kaplan–Meier estimates of progression-free survival (A) and overall survival (B) are shown for the following 4 treatment groups: metformin group, non-metformin group, discontinued group, and nondiabetic group.

**Figure 2:**
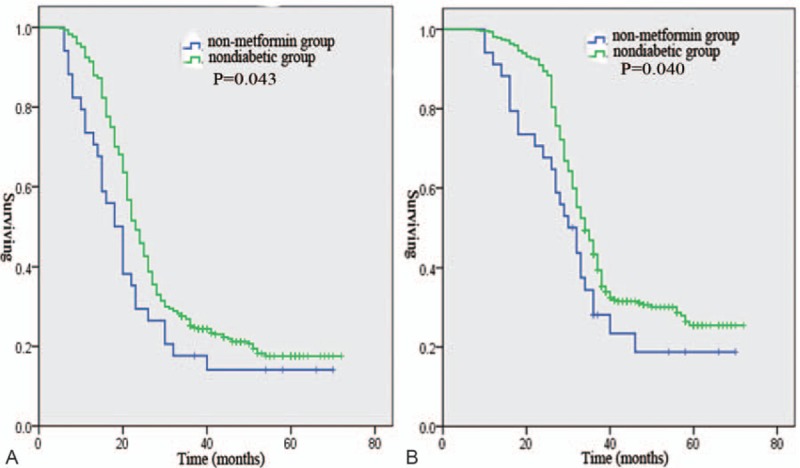
Progression-free survival (A) and overall survival (B) of ovarian cancer patients in the non-metformin group and the nondiabetic group.

**Figure 3:**
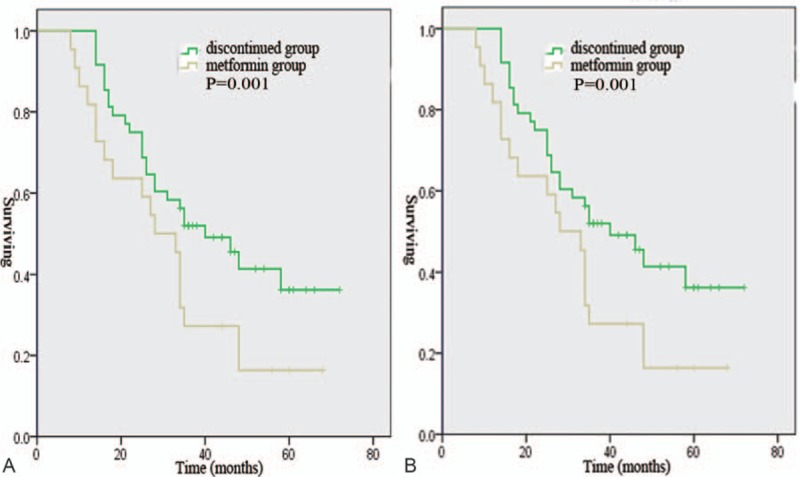
Progression-free survival (A) and overall survival (B) of ovarian cancer patients in the metformin group and the discontinued group.

**Figure 4:**
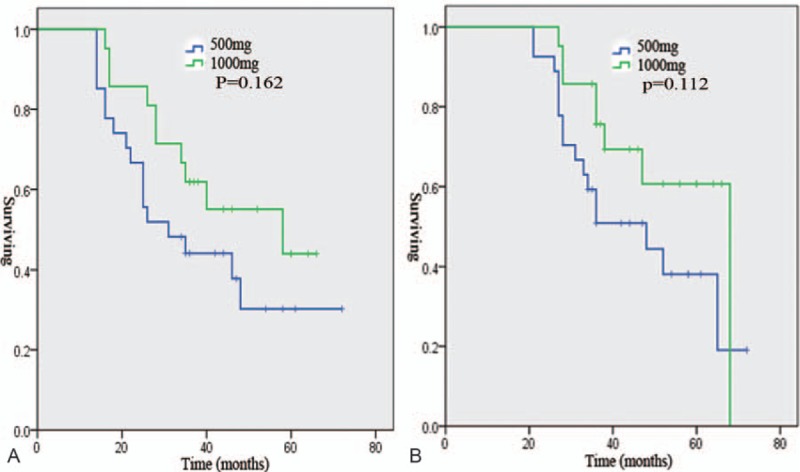
Progression-free survival (A) and overall survival (B) of diabetic patients treated with 500 mg metformin twice daily and with 1000 mg metformin twice daily.

There were no differences in the treatment strategies among the 4 groups. However, a longer median PFS was observed in the metformin group than in the non-metformin group, the discontinued group, and the nondiabetic group (40 vs 18.2 vs 28 vs 23.3 months, *P* = .001, Fig. 1A). A longer median OS was observed in the metformin group than in the non-metformin group, the discontinued group, and the nondiabetic group (52.1 vs 30 vs 32 vs 34.2 months, *P* = .007, Fig. 1B). A shorter median PFS was observed in the non-metformin group than in the nondiabetic group (18.2 vs 23.3 months, *P* = .043, Fig. 2A). A shorter median OS was observed in the non-metformin group than in the nondiabetic group (30 vs 34.2 months, *P* = .04, Fig. 2B). Moreover, patients in the discontinued group had a significantly poorer median PFS (28 vs 40 months, *P* = .001, Fig. 3A) and OS (32 vs 52.1 months, *P* = .001, Fig. 3B) than patients in the metformin group. In the metformin group, a similar PFS (Fig. 4A, *P* = .162) and OS (Fig. 4B, *P* = .112) were observed between diabetic patients treated with 500 mg twice daily and diabetic patients treated with 1000 mg twice daily.

After adjusting for the possible confounders in the multivariate Cox proportional hazard model, similar survival rates were observed among the treatment groups. Compared with diabetic patients who did not use metformin, the diabetic patients who used metformin had an obviously decreased risk for disease relapse [hazard ratio (HR) = 0.37; 95% confidence interval (CI): 0.24–0.58; *P* < .01] and disease-related death (HR = 0.43; 95% CI: 0.23–0.66; *P* *=* .02) (Table 2). The discontinued group also had a decreased risk for disease relapse (HR = 0.88; 95% CI: 0.48–1.72; *P* *=* .45) and disease-related death (HR = 0.91; 95% CI: 0.64–1.87; *P* *=* .74), but this difference was not statistically significant. Moreover, compared with patients in the nondiabetic group, diabetic patients who used metformin, but not those who discontinued metformin use, had increased PFS (HR = 0.34; 95% CI: 0.27–0.67, *P* < .01) and OS (HR = 0.29; 95% CI: 0.13–0.58; *P* *=* .03) (Table 2).

**Table 2:**
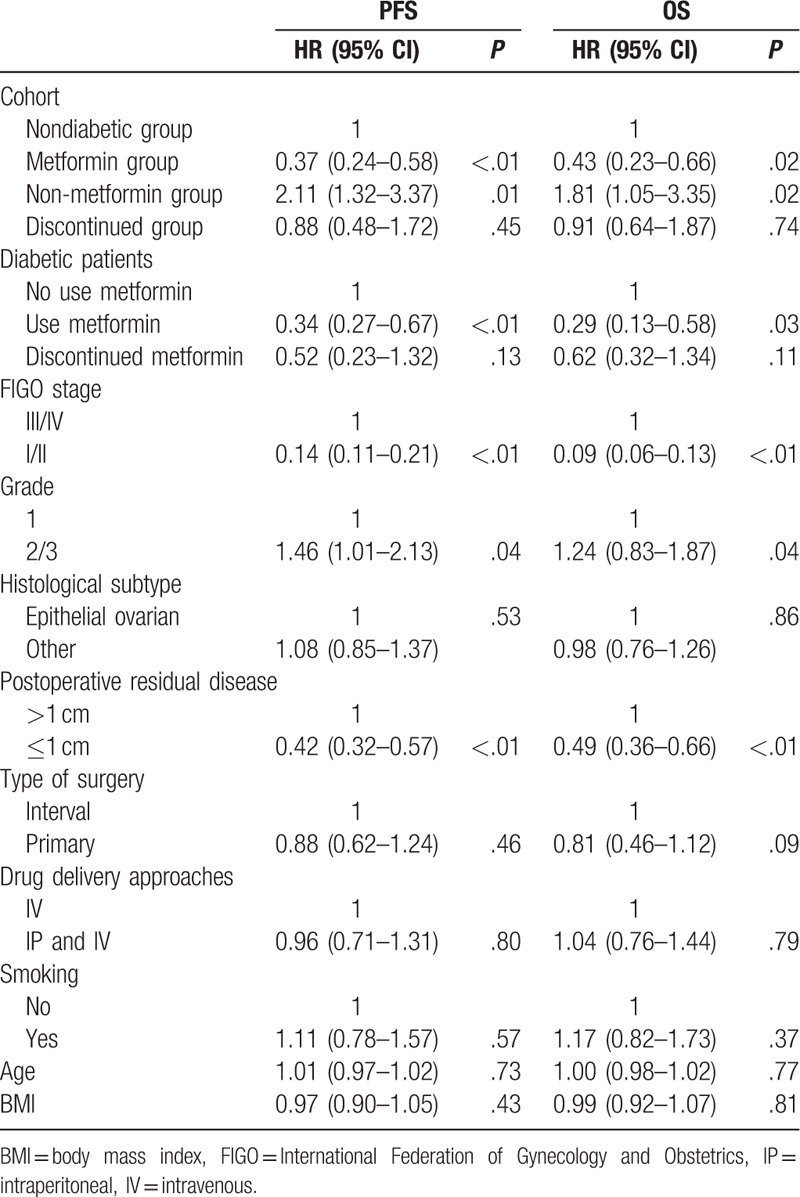
Multivariable Cox mode for progression-free survival and overall survival.

## 4 Discussion

In the present retrospective cohort study, we observed that type 2 diabetic patients with ovarian cancer who used metformin had a longer PFS and OS than type 2 diabetic patients with ovarian cancer who did not use metformin. Compared with ovarian cancer patients without diabetes, diabetic patients who used metformin had longer PFS and OS. The multivariate Cox proportional hazard model showed that metformin use was associated with a lower risk for disease relapse (HR = 0.34; 95% CI: 0.27–0.67; *P* < .01) and disease-related death (HR = 0.29; 95% CI 0.13–0.58; *P* *=* .03) among ovarian cancer patients with diabetes. These findings are consistent with those of previous studies.^[20,21]^

Metformin has been commonly used as a first-line pharmacotherapy for type 2 diabetes and is a well-tolerated antidiabetic drug. In vitro and in vivo experiments have shown that metformin has anticancer effects.^[24,25]^ Although the mechanism underlying its anticancer effects is uncertain and requires additional research, many in vitro studies have proposed that metformin suppresses hepatic gluconeogenesis, protein synthesis, and the proliferation of cancer cells by adenosine monophosphate activated protein kinase (AMPK) activation and suppression of the mammalian target of the rapamycin signaling pathway.^[8,24,26,27]^ Moreover, metformin may decrease circulating insulin levels and partially reverse insulin resistance induced secondary hyperinsulinemia,^[28,29]^ which leads to the inhibition of carcinogenesis and/or cancer cell growth.

Patients with type 2 diabetes are insulin resistant and hyperinsulinemic, which lead to increased systemic insulin levels. Previous studies have suggested that increased insulin levels are associated with a high risk of disease recurrence and death in patients with ovarian cancer.^[20–22]^ In the present study, similar results were observed in type 2 diabetic patients who did not use metformin. However, diabetic patients who used metformin had longer PFS and OS than ovarian cancer patients without diabetes and diabetic patients who did not use metformin. After adjusting for age, BMI, smoking, FIGO stage, pathological type, pathological grading, postoperative residual disease, type of surgery, and drug delivery approaches, we found that metformin is an independent predictor of survival among ovarian cancer patients.

Many in vitro studies have reported that AMPK/mammalian target of rapamycin (mTOR) are associated with the noninsulin-dependent cytotoxic effects of metformin at concentrations ranging from 5 to 10 mmol/L.^[26,30–32]^ When the concentration of metformin was lower than 5 mmol/L, few studies detected cytotoxic effects from metformin treatment.^[32]^ Therefore, some authors believe that the anticancer effects of metformin are dose dependent.^[32–34]^ However, there was no significant difference in survival between type 2 diabetic patients who took high-dose metformin (1000 mg twice daily) and type 2 diabetic patients who took low-dose metformin (500 mg twice daily). Moreover, one interesting finding was that type 2 diabetic patients who continually used metformin exhibited significantly better survival than diabetic patients who discontinued metformin use. In vitro studies have claimed that metformin suppresses the growth of cancer cells and promotes cell cycle arrest in the G0/G1 phase but does not induce cancer cell death.^[24,26,35]^ Therefore, when diabetic patients discontinue metformin treatment, the dormant ovarian cancer cells start to proliferate, leading to disease recurrence. In addition, the growth of cancer cells in vivo can be inhibited by an antidiabetic dose of metformin.^[36,37]^ Thus, a lower dose of metformin may decrease the risk of disease recurrence and death in patients with ovarian cancer, but metformin use still needs to be sustained for treatment efficacy. To the best of our knowledge, this is the first study to show that diabetic patients with ovarian cancer who discontinued metformin treatment have an obviously increased risk of disease recurrence and death compared with patients who continued metformin treatment.

Although the current study has shown the positive effect of metformin on survival among ovarian cancer patients with diabetes, the study is limited by the retrospective design and small sample size. In addition, in the present study, we cannot completely exclude the possibility that metformin intake may be associated with other prognostic factors, such as insulin levels, glycosylated hemoglobin A1C levels, and thiazolidinedione use. To overcome these limitations, large-scale prospective trials are required.

In conclusion, ovarian cancer patients with type 2 diabetes who continue taking metformin exhibit greater survival rates than those patients not taking metformin.
